# Supplementary material for: Analysis and Risk Assessment of Pesticide Residues in Strawberry Using the PRIMo Model: Detection, Public Health and Safety Implications
Source: Foods. 2025 Oct 11;14(20):3470. doi: 10.3390/foods14203470 (PMC12563036; doi:10.3390/foods14203470)
Supplement: Supplementary file 1 [file foods-14-03470-s001.zip › foods-3891090-supplementary2.pdf]

Analysis and Risk Assessment of Pesticide Residues in Strawberry using the PRIMo Model: Detection, Public Health and Safety Implications

Elvira De Rosa[1,2], Maddalena Di Lillo[1], Maria Triassi[1,2], Fabiana Di Duca[1]*, Immacolata Russo[3], Vito Graziano[1], Giovanni Mazzei[1], Immanuela Gentile[1], Seyedeh Zahra Shojaeian[1], Paolo Montuori[1]

1. Department of Public Health, "Federico II" University, Via Sergio Pansini nº 5, 80131 Naples, Italy
2. Department of Human Sciences and Quality of Life Promotion, San Raffaele University, 00166 Rome, Italy
3. Department of Public Health, "Federico II" University Hospital, Via Sergio Pansini nº 5, 80131 Naples, Italy

* Correspondence: fabianadiduca91@gmail.com

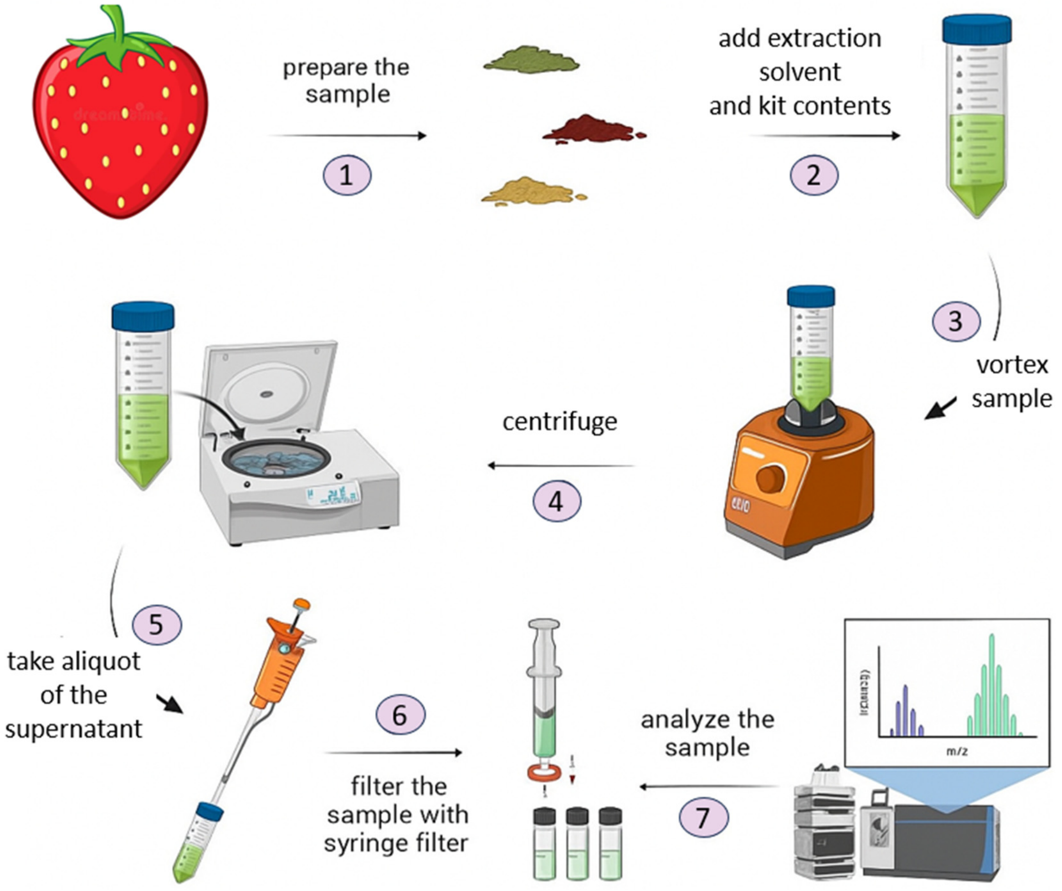

Figure S1. Schematic representation of the operational steps of the modular QuEChERS method (UNI EN 15662:2018) for the extraction and purification of pesticides from food matrices.

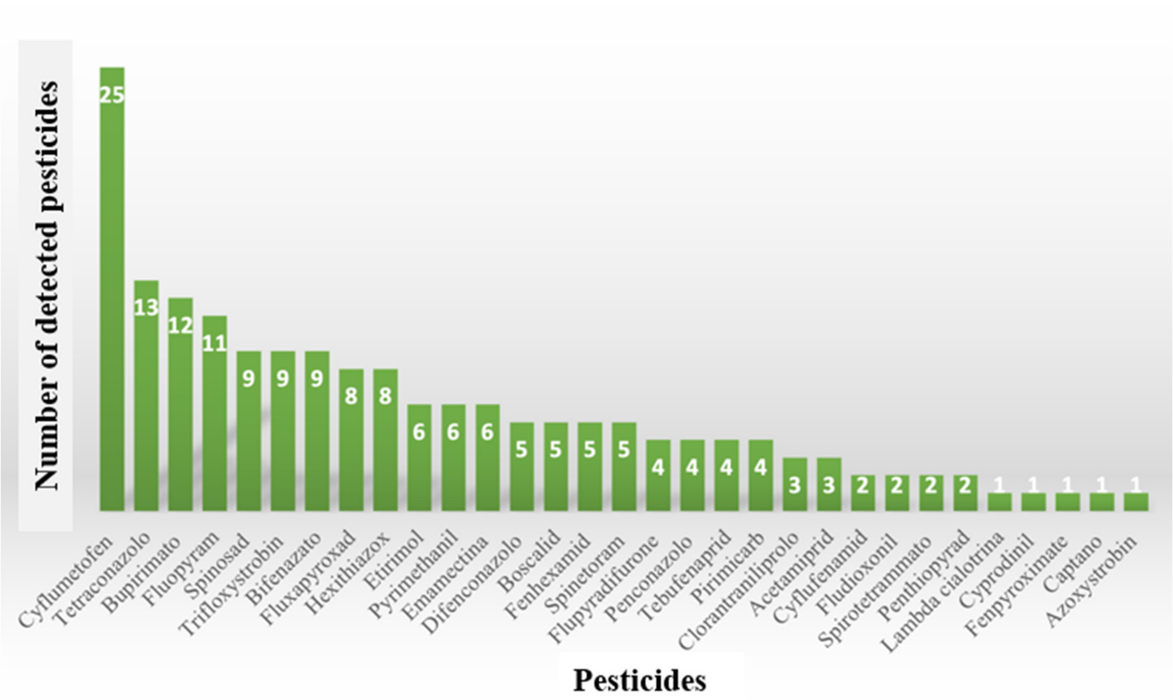

Figure S2. Distribution of the number of samples with detected pesticide residues.

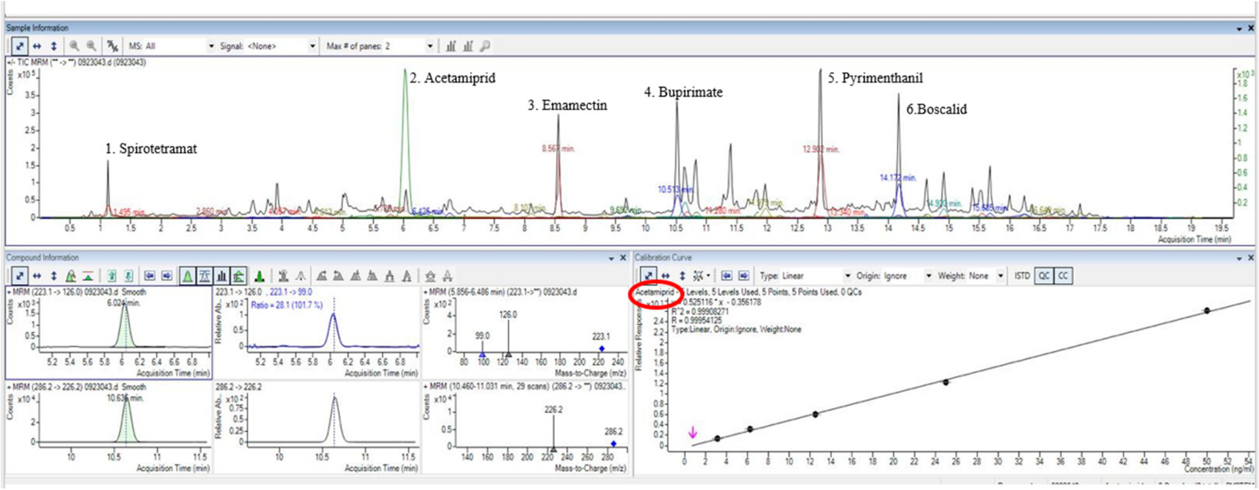

Figure S3. Representative chromatogram of the pesticides analyzed in strawberry samples. The following compounds are indicated in detail: 1) Spirotetramat, 2) Acetamiprid, 3) Emamectin, 4) Bupirimate, 5) Pyrimethanil, and 6) Boscalid. The mass spectrum of Acetamiprid is also shown.

Table S1 “Characteristic ion peaks and validation parameters for the qualitative and quantitative analysis of pesticides”.

Pesticide	Precursor (Q1 m/z) → Product ions (Q3 m/z)	Mean±SD	LOQ (mg/Kg^{-1})	RSD (%)	Recovery (%)
Hexythiazox	353.1 → 168.0; 353.1 → 227.7	0.0390 ±0.0051	0.010	13.0	96
Cyflumetofen	448.2 → 173.0; 448.2 → 145.0	0.1120±0.0058	0.010	5.2	90
Tebufenaprid	334.2 → 117.0; 334.2 → 145.0	0.0730±0.0066	0.010	9.1	91
Bifenazate	301.1 → 198.0; 301.1 → 170.0	0.0400±0.0039	0.010	9.8	91
Fenpyroximate	422.2 → 366.1; 422.2 → 138.1	0.0790±0.0042	0.010	5.4	97
Bupirimate	317.0 → 166.1; 317.0 → 108.0	0.1260±0.0062	0.010	5.0	96
Fluxapyroxad	380.1→ 50.0; 380.1 → 48.0	0.2670±0.0054	0.010	2.0	91
Difenoconazole	489.2 → 316.1; 489.2 → 208.1	0.1150±0.0054	0.010	4.7	81
Cyflufenamid	413.1 → 310.1; 413.1 → 131.1	0.0150±0.0019	0.010	13.0	85
Penconazole	284.1 → 158.0; 284.1 → 70.0	0.0350±0.0058	0.010	16.7	81
Cyprodinil	226.0 → 93.1; 226.0 → 77.0	0.2600±0.0046	0.010	1.8	81
Fludioxonil	247.0 → 180.0; 247.0 → 152.0	0.1250±0.0066	0.010	5.3	83
Etirimol	210.0 → 140.0; 210.0 → 182.0	0.0250±0.0019	0.010	7.8	94
Pyrimethanil	200.1 → 51.0; 200.1 → 50.0	0.6340±0.0066	0.010	1.0	91
Fluopyram	397.0 → 208.0; 397.0 → 173.0	0.0650±0.0070	0.010	10.8	86
Trifloxystrobin	409.1 → 186.0; 409.1 → 131.1	0.0520±0.0074	0.010	14.3	96
Tetraconazole	372.1 → 89.0; 372.1 → 72.9	0.0340±0.0039	0.010	11.5	98
Boscalid	343.1 → 306.8; 343.1 → 139.9	0.0590±0.0046	0.010	7.9	96
Fenhexamid	302.1 → 97.1; 302.1 → 143.0	0.0990±0.0062	0.010	6.3	92
Captan	300.9 → 264.9; 300.9 → 151.9	0.0300±0.0058	0.010	19.5	84
Penthiopyrad	358.0 → 149.0; 358.0 → 109.0	0.1240±0.0050	0.010	4.1	93
Azoxystrobin	404.1 → 372.1; 404.1 → 344.1	0.0120±0.0019	0.010	16.3	95
Spinosad	733.0 (A); 747 (D)	0.0300±0.0027	0.010	9.1	94
Chlorantraniliprole	484.0 → 453.0; 484.0 → 285.8	0.0110±0.0019	0.010	17.7	93
Flupyradifurone	289.0 → 126.0; 289.0 → 90.0	0.1950±0.0058	0.010	3.0	93
Lambda-cyhalothrin	449.0 → 197.0; 449.0 → 181.0	0.0150±0.0023	0.010	15.6	90
Spirotetramat	374.2 → 330.2; 374.2 → 302.2	0.1960±0.0062	0.010	3.2	86
Spinetoram	748.4 → 142.0; 748.4 → 98.1	0.0320±0.0019	0.010	6.1	96

Pirimicarb	239.15 → 72.1	0.0920±0.0066	0.010	7.2	94
Emamectin	890.5 → 305.1; 890.5 → 567.2	0.0220±0.0023	0.010	10.6	95
Acetamiprid	223 → 126; 223 → 56	0.0190±0.0031	0.010	16.4	96

Table S2 “Chromatographic and Mass Spectrometric Conditions”

**Parameter**	**Value / Conditions**
Instrument	Agilent 6495C triple quadrupole LC/MS
Column	Acclaim C18, 4 µm, 250 × 4.6 mm
Mobile Phase A	Water/Methanol 95:5 + 0.1% formic acid / 5 mM ammonium formate (LC-MS grade)
Mobile Phase B	Methanol/Water 95:5 + 0.1% formic acid / 5 mM ammonium formate (LC-MS grade)
Gradient	5% B (0 min) → 95% B (10 min) → 95% B (13 min) → 5% B (13.01 min)
Column Temperature	25°C
Flow Rate	0.3 mL/min
Injection Volume	2 µL
MS/MS Mode	SRM (Selected Reaction Monitoring), ESI positive
Capillary Voltage	4000 V
Fragmentor Voltage	190 V
Drying Gas	9 L/min at 325°C
Collision Gas	Medium
Nebulizer / Auxiliary Gas	45 psi each
Software	Mass Hunter (Applied Biosystems)
